# The Use of Social Media to Combat Research-Isolation

**DOI:** 10.1093/aesa/sax051

**Published:** 2017-09-06

**Authors:** M. A. Reeve, M. Partridge

**Affiliations:** 1 Institution of Environmental Sciences, Floor 3, 140 London Wall, London, EC2Y 5DN, United Kingdom (michelle.a.reeve@gmail.com); 2 Corresponding author, e-mail: michelle.a.reeve@gmail.com; 3 Centre of Engineering Photonics, Cranfield University, Cranfield, Bedfordshire MK42 0AL, United Kingdom (m.c.partridge@cranfield.ac.uk)

**Keywords:** social media, isolation, mental health, network, research

## Abstract

Research-isolation is a common problem affecting many researchers who are disconnected from their research communities. It can be caused by a number of factors, including physical isolation, unfamiliar research topics, diversity, and the nature of the supervisory relationship. All of these aspects can have an impact on both work and the mental health of researchers. Increasingly, researchers are turning to social media for support, by both looking for communities and for increasing the impact of their work. In this paper, we set out a brief introduction to a range of social media platforms used by researchers and present a discussion of the networks within those platforms aimed at reducing research-isolation. These examples highlight just a few of the number of small communities that have grown online to meet the needs of those seeking support through social media. We conclude with some recommendations for those affected by research-isolation and highlight the need for more research into the role of social media on mental health in academics.

Isolation is commonly typified by either the inability of or unwillingness to take part within the community around one’s work, including both the local social community but also the wider research community which a researcher may feel unable to access. Research-isolation is a very real problem in research institutes. Research-isolation is here used as a term that includes both social disconnect and perceived isolation (Cornwell and Waite 2009) of researchers in both academia and industrial settings. This impacts not only the research itself, but many other aspects of a researcher’s life, and can also negatively affect mental health, especially if prolonged (Shaw and Ward 2011, Shaw 2014). Finding new methods of communication to break down isolation can provide a solution to this issue.

Social media is one such communication tool that has impacted a wide range of areas from politics (Loader and Mercea 2011) to teaching (Tess 2013). Over the past 10 yr, social media use has grown from 7% to 65% of adults worldwide (Perrin 2015). The way people communicate and their networks are changing rapidly, and academic research is just one of the areas affected by these trends (Bik and Goldstein 2013).

A wide array of social media platforms are available, all with different functions and aims. These cover a number of useful roles for researchers: communicating their work, searching for jobs, networking for potential collaborators, or asking for advice. Researchers often work in groups and are therefore surrounded by peers, relying on them and their institution for advice, networking, and general support. Many of the functions provided by social media can of course be fulfilled by these laboratory colleagues and supervisors, or management structure. At an institutional level, support is often focused on early career researchers, as this period can be a particularly stressful for the individual. Support in this sense can comprise of counseling sessions or assigning mentors. Peers and supervisors can introduce researchers to collaborators to expand their network, either at conferences or during laboratory visits, and also help early career researchers find postdoctoral and postgraduate positions.

Not every researcher has this experience as part of a research group. Many simply do not have, or are not comfortable accessing, those kinds of internal support networks, for a variety of reasons (Marshall etal. 2010). Some institutions may be lacking in support facilities, or researchers may be working in a very small laboratory group where networking and opportunities for advice are more limited. Researchers working remotely owing to necessity, such as completing fieldwork or through disability or illness, may miss opportunities for engagement with their laboratory group, and upon their return, this may cause them to may feel separated from their peers. Individuals who may feel isolated owing to being a different age, race, gender, or academic level to those around them might find it harder to reach out for support, even if it is available. All of these examples can lead to cases of research-isolation.

Here, we discuss four key causes of research-isolation: physical isolation; research topic, diversity, and the supervisory relationship. This list is by no means exhaustive, and each cause can contribute independently or collectively to these feelings of isolation (Shaw 2014, The Thesis Whisperer 2015).

Perhaps the clearest of causes is that of physical isolation, where the laboratory, university campus, or research institution is located far away from other researchers. Physical isolation often directly affects the more social side of a researcher’s life, and, especially if they live in or close to the isolated area (Golden etal. 2008). Physical isolation can also occur through extended home-working, for example during thesis writing, through illness, or new parenthood (Moss 2016). Physical isolation can leave researchers feeling out-of-the-loop or sidelined compared with their peers within the same research area.

However, even when physically located within a group, working on a different topic to those around them can in itself cause isolation. In this instance, the individual may be limited in who they can ask for subject-specific advice, and may not have the “sounding board” that lots of researchers have in their colleagues. Less experienced early career researchers may be particularly affected in this situation, as it is reasonable to suggest that they would require more time to compile literature reviews and plan experiments than would be needed for others who are able to draw on knowledge of their peers working in a similar area. This can introduce the feeling of being behind in their work, leaving the researcher feeling particularly detached from those around them.

In addition to physical or topic isolation, diversity can be an isolating issue in research (Smith and Calasanti 2005), as it is in many industries. Those individuals who are in the minority within a laboratory group, be that because of gender, race, sexuality, age or disability, or even political preference, can feel isolated and alone (Powell 2007, Nivet 2010). Zimmerman etal. (2016) found that women in academia report being ostracized in the workplace more frequently than men and that faculty members of color report more frequent information exclusion, defined as “situations where people perceive being uninformed of information known mutually by others” (Jones etal. 2009), than white faculty members. Some diversity groups can be affected at different stages of academic careers; for example, it is well-reported that women hold fewer senior researcher or professor roles than men (EU 2009; van den Brink and Benschop 2012; Institute of Medicine, National Academy of Sciences and National Academy of Engineering 2007), which could contribute to isolation. New or single parents can also be isolated owing to difficulties of being able to attend professional networking events, such as conferences (Anonymous 2017).

Finally, a key cause of isolation can be the quality of the student–supervisor relationship during early academic career stages. There is anecdotal evidence (often anonymous) which suggests that a suboptimal supervisory relationship can play a large role in exacerbating feelings of inadequacy and isolation. A supervisor’s leadership style has even been cited as one factor which can negatively impact PhD students’ mental health (Levecque etal. 2017). For example, supervisors who fail to recognize stress (Anonymous 2014), have different working styles to the student (Anonymous 2013), or who give inappropriate, or minimal feedback on work (Anonymous 2015) can make an early career researcher unsure of themselves, and reluctant to reach out for support, as doing so can often be perceived as weakness (Anonymous 2015).

These are just a handful of examples of potential causes of research-isolation. Many personal situations can also contribute to feeling isolated, such as the previously discussed new parenthood, illness, or disability, and sudden traumatic life events, for example serious illness or death of family members. Events such as this can affect academic performance and productivity (Gertler etal. 2004, Servaty-Seib etal. 2006, Beach 2014), as well as life outside work.

Research-isolation can affect many aspects of an academic’s life. No matter why the isolation is occurring, if support networks are limited, then the work itself can suffer. More importantly, prolonged research-isolation can have real implications on mental health (Gewin 2012, Shaw 2014, Breines 2015). Academia is typically an industry where people work long, intense hours (Jacobs and Winslow 2004); though the myths of needing to work 80-h weeks are now being challenged (Duffy 2015), it is still prevalent, and when coupled with isolation, can cause a lot of undue pressure.

Researchers are increasingly using social media (Collins and Hide 2010) as a way of combating isolation. Social media provides an easily accessible means of communicating their work to a wider and interested audience (Bukvova etal. 2010, Brossard and Scheufele 2013), discussing experimental problems and asking for advice, and even for recruiting subjects for experiments (O’Connor etal. 2014, Shere etal. 2014, Yuan etal. 2014).

In this paper, we set out a brief introduction to a range of social media platforms used by researchers and present a discussion of the networks within those platforms aimed at reducing research-isolation.

## Social Media Platforms

**Fig. 1.**
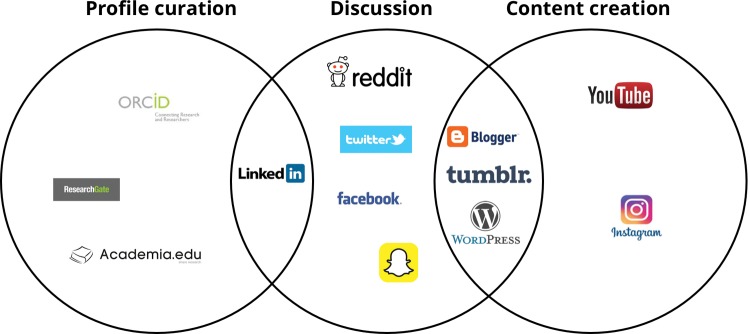
Venn diagram showing the overlap between the various research-focused social media platforms.

The growth in social media in the past decades has generated numerous services, all of which have varying methods for social interaction. They are constantly changing and evolving; services that were built with a narrow focus and structure have often changed beyond all recognition. Here, we present a number of the currently available platforms as a summary of the state-of-the-art in social media. A visual summary can be seen in Fig. 1. We also examine the use of these services in a research context, and how they might be used to prevent or confront research-isolation.

### Profile Curation

In the context of social media, we use profile curation as a term to define platforms, which are primarily a way of hosting a public profile of the user. They differ to discussion (section “Discussion Platforms”) or content-based (section “Content Creation”) services, which are more reliant on a steady stream of updates provided by the user. Typically, the most basic information hosted on these services consists of name, employment, and publications. These act as virtual CVs, which are often linked to by other services.

Profile curation social media requires the lowest frequency of updates, yet still acts as a valuable tool to provide a central place where others can find individuals and their professional details. As a result, use of curation kind of social media can lead to external network building and collaboration. Some universities have begun requiring researchers to sign up to one or more of these services, and some have used these to replace their own profile database entirely (Anonymous 2016c).

#### ORCID

Open Research ID, or ORCID, is a profile curation social media service specifically aimed at academics. It allows users to host information on employment history alongside various types of publications and funding. There is no networking, and only a single, optional biography box to discuss a user’s work in more context. The ethos of ORCID is to just provide the important information in a simple to read easily accessible format.

One of the great strengths of ORCID has been the growing integration with other services so that many of the fields can be populated automatically. In particular, integrating with larger paper cataloging services, such as the SCOPUS publication library, means that the curation aspect of ORCID is to some extent automatic. ORCID has also integrated with many journal services and some even require an ORCID ID. Recently, ORCID has also started integrating with other services commonly used in universities, such as data access systems (e.g., figshare), and with university profile systems (e.g., Current Research Information System or CRIS). ORCID’s ability to automate data sharing between all these services makes it a very popular system, with currently over 3 million users (ORCID 2017).

#### Academia.edu

On the surface, Academia.edu looks like an expanded equivalent of ORCID. It has many of the same features and allows users to list their job, publication list, and employment history. Academia.edu allows for more network-style features, and it will link the details that users add with other researchers so that users can, for example, see all other people listed as employees of a particular employer. The other key difference from ORCID is in the way it handles publications. Academia.edu wants to host publications directly within its servers and will prompt users to upload these. These are only available to other users of Academia.edu. The networking updates are also driven by new uploads, so any paper that users repost on Academia.edu will be shown to anyone in their network. It should also be noted that Academia.edu is run with a different model to ORCID and has come under significant criticism because of the way it allows researchers to promote themselves within its search system (Bond 2017).

#### ResearchGate

ResearchGate is similar to Academia.edu. Again, it allows users to have a static academic profile showing off their employment history and publications, and like Academia.edu, it asks for copies of research papers which it can then make available to anyone looking at the author’s profile. However, the networking opportunities on ResearchGate are much wider, and there is more emphasis on engagement with other people. In particular, there is the ability to post public questions either asking for help within a topic or even just to a particular researcher. Questions allow people to connect more easily both with colleagues but also other academics. The focus on connecting to people, as well as the suggestions to contact other academics to ask questions or for copies of papers, does contribute to users actively engaging with each other.

#### LinkedIn

With 100 million users (Anonymous 2016a), LinkedIn is one of the largest profile curation platforms, and is aimed at professionals rather than specifically at researchers. Like ORCID, it provides a public page showing some basic information on a user as well as their job history and publications. LinkedIn, however, goes further and provides more rich text areas for discussion of a user’s work and publications so that users can see them in context. The resulting page is a profile which reads as a fuller CV. In addition, LinkedIn also acts as a networking tool and has several features specifically aimed at connecting its users. A user can add people to a network and categorize these people as colleagues, friends, etc. From these connections, LinkedIn will then produce updates and suggestions of new connections users might wish to make from the existing people in their network. LinkedIn also provides news feeds akin to Facebook for users to publish updates and comment on other users’ pages. These news feeds offer an opportunity to engage with others through their work and subsequently encourage interaction with the user.

### Content Creation

There are a variety of social media platforms which can be used for creating content about the researcher and their research, to engage with others or simply communicate complex work. The type of content produced depends on the user’s preference, and also to some extent, the nature of the research itself—is it visually interesting, or perhaps, it requires in-depth explanations? Different services cater to these categories more readily, with options depending on what media format users prefer. Some networks have more a supportive nature, supporting each other as academics, often by using certain hashtags or by interacting with groups which have been created and are run specifically for the purpose of sharing content.

Here, we describe a number of social media services which many researchers use for communicating their work, engaging with others, and seeking support, all of which are useful in combating research-isolation.

#### YouTube

YouTube, a platform for videos, is one such site which is suitable for more visually engaging work. Video format is ideal for translating more complex scientific concepts relating to a user’s research, but requires often expensive equipment and software to film and edit. Perhaps for this reason, it tends to be institutions which produce video content rather than the individual researcher, often as part of promotion for particular events, such as science festivals or open days. It can also take a lot of time to plan, film, and edit video content, which is another reason why a typically busy researcher may not wish to pursue video-based communication. Time spent on video creation can be a good investment; however, over 1 billion people use YouTube, watching over 3 billion h of video every month (Anonymous 2016d).

#### Instagram

Instagram is primarily a photo-sharing site which allows users to take and edit photos using filters and other image settings. Photo-sharing is a rapid way of sharing something visual about a user’s research. It also provides a visual insight into other research environments, which is an advantage over text-based platforms (Pittman and Reich 2016). Popular and common things to share include behind-the-scenes photos of methods or technology used, such as a 3D printer mid-print or unidentifiable experimental equipment. Instagram also allows users to share short videos (up to 60 s; Lee 2016), and have recently introduced a new “Stories” feature, where users can share a slideshow of short videos or images that disappears from their feed after 24 h. Richer image and video footage can be a good way to share a research method or concept which ideally requires more than one image to explain, allowing followers to view the images or clips in the intended order, without then clogging up users feed. Hashtags can be used to signpost a particular field or area of science, as well as tapping in to the academic network on Instagram itself.

#### Blogs

Blogs are typically presented in written form, and are a way of engaging and communicating research with other scientists. There are numerous blogging sites available, such as WordPress and Blogger, which in a research context are primarily used for longer-form written pieces interspersed with a few images, either about the research itself, or about more personal experiences of academia. There are also other blogging sites which are predominantly suited to sharing media content, such as videos, GIFs, and even musical pieces. A good example of this kind of mixed media site is Tumblr.

Many researchers choose to start a blog as a way of practicing and developing their writing skills (Kjellberg 2010), especially early career researchers such as PhD students who may not have as much writing experience. If writing about personal experiences of academia, it can also be an effective way of engaging with others in similar positions, creating support networks, as well as often being cathartic for the writer. Depending on a researcher’s writing skills, blogging can be a quick way of generating content for others to engage with, though perhaps it is eclipsed by the large number of science bloggers producing similar content, making it more difficult for other users to find a user’s content just by chance.

### Discussion Platforms

Discussion social media platforms are, unlike profile curation and content creation platforms, more aimed at being places for dialog and comment. These typically consist of regular short-form informal updates and resharing of interesting content for discussion. Being shorter and less formal means that replies and feedback on questions and comments is often quicker than other network and from a wider range of sources. There are also a number of research communities that make active use of these platforms.

#### Facebook

Facebook is the world’s largest social media network, with almost 2 billion users (Anonymous 2016b). It allows users to upload some personal information as well as professional information about employment and education. It is designed as a social media network for everyone and so lacks some of the research-specific features of ResearchGate, ORCID, or Academia.edu, such as adding publications and funding. There is also more emphasis on status updates and discussion of current stories on a user’s news feed. Users typically update their feeds in the range of anything from once a month to multiple times daily (Duggan etal. 2014).

Facebook also has an increasing emphasis on providing a social space with customizable privacy. Users can create a range of groups (e.g., family, coworkers), then customize who can see which of their updates. Customization can be a good way to ensure the right people see a user’s updates and can help maintain a professional profile separate to a personal profile on the same network.

As it is more broadly focused, Facebook is not often considered a “professional” platform but rather a personal platform. Nonetheless, it is increasingly being used for professional networking and discussion of research, as discussed in section “Networking through social media”. It is also a key part of disseminating any content such as images and videos owing to its broad reach.

#### Twitter

Twitter is a social platform that is focused on short (140 characters) status updates called Tweets. Users may upload short biographies on their profile, but the biographies are also limited to just 160 characters, and focuses on simply identifying the users. Many users stay anonymous on Twitter, and it is not always expected that users identify themselves.

The updates can be anything that fits the 140 character format and can include photos, videos, and links. Perhaps owing to their short length, the frequency of updates on Twitter is typically higher than other platforms, with many people tweeting 10–20 times per day (Java etal. 2007). Some people instead choose to use Twitter purely as a passive news feed to keep updated on people working in their field. The focus of updates varies from people sharing stories of their research, commenting on news articles, to discussions about recipes.

Twitter also relies on the resharing of other people’s status updates via Retweeting. Resharing can mean that even a user with a limited number of followers can quickly reach thousands of people. As discussed in section “Advice,” there are a number of support groups and specifically research-focused hashtags on Twitter (Scobble 2016).

#### Reddit

Reddit is different to other social media networks in a number of ways. Firstly, it is rare to find anyone publicly identified, as most users will be anonymous. Secondly, the network is highly variable in terms of user experience. Reddit is a collection of subreddits, each focusing on a narrow range of topics. In each subreddit, people can post links, images, or videos which are then voted up and down and commented on. Comments can also be voted up and down. The voting system has the effect of pushing certain comments or posts to the top of the feed, making them more visible. Each subreddit has its own rules about what can be posted and the kind of comments that are allowed.

All of these subreddits are community-managed and range from “worldnews” to “catstandingup.” Some communities can be, by design, hostile places to contribute. However, Reddit is as diverse as the rest of the Internet, and there are communities that are less tolerant to any abuse and very open to new members. There are some key research communities discussed later in section “Advice,” but there are a great many subreddit communities on Reddit, and moving beyond the default ones and searching for subreddits that match the sort for information or communities a user wants to be a part of is very important.

#### Online Forums

Forums are one of the longest standing forms of social media and predate almost all of the other platforms. There is some debate as to whether or not they can be defined as social media (Weinberg 2008), but within the context of researcher-isolation, they are certainly a social platform that can have many of the same features and benefits of other social media.

Forums are usually hosted websites where users login to post on to a message board, typically organized by topic. Many are open to read, but posting on them requires an account. Forums are simple to set up and most are comparatively small communities with a particular focus. Different forums have different rules about the type of content that can be posted, but typically they are text-based.

These communities tend to be based on a conserved membership of people contributing and will have very loyal users. They are also usually moderated by a select number of users to make sure comments are on topic and not abusive.

## Networking Through Social Media

The previous section dealt primarily with the provision of social media services by companies and organizations. These are all services which provide the platform on which to build a social network. However, one of the great strengths of social media for researchers is not just in the platforms and their various pros and cons, but in the networks and support available through them. These networks can simply be a support network that grows slowly through contacts and friends, but within all of these services, there are already projects specifically aimed at being a support network for researchers.

Here, we describe a selection of these networks chosen to illustrate the availability of support. It is not a comprehensive list of these groups available. In this section, we have categorized these into loose areas of focus; yet, much like the platforms they use, there are networks that span multiple categories.

### Research Support

Research support groups and networks are places that actively work to provide either collective services or research-targeted support. In many cases, these are run or curated by other researchers, hoping to promote good research habits and networks. There are a wide number of networks, ranging from assisting with mental health to even helping to find the best paper-authoring software. Most communities are open and do not require any membership to either participate or read the available help. In almost all cases, actively participating can be more rewarding, as the user can ask specific questions or for specific help based on their own experiences and situation.

Support groups like the ones described below provide two services which are important for mental health. Firstly, they can give isolated researchers support, which they may not be able to get more locally. As discussed, student supervision and teaching can be variable, and these groups provide critical help that students can search for themselves. Secondly, the groups are often places that encourage discussion and sharing of problems, which can provide researchers with insight into the wider community and a sense of inclusivity with others facing similar problems.

#### Shut Up and Write

Shut up and Write was founded to encourage researchers to overcome issues with writing, which many find difficult. Started in 2013 by the Thesis Whisperer (The Thesis Whisperer 2013), it spans across many social media platforms, and is an event where academics either come together physically or remotely at an agreed time and write as much as possible in a given time span. Since its inception, it has been expanded and copied by many organizations, and several universities now have Shut Up and Write nights in the libraries. Through Twitter, there is also an account which prompts researchers to do a fortnightly hour-long Shut Up and Write session, and facilitates discussions before and after the event to talk about writing tips and advice.

#### Online Journal Clubs

Journal clubs are a common local support system, which are often forums where students can discuss relevant papers and keep up-to-date with the literature. The provision of journal clubs is sporadic, however, depending on the institute and size of the research group. Social media has allowed journal clubs to expand from being a local support network to being a wider online community support network. Researchers joining a journal club can sign up to read or propose a paper that all those in the group then discuss via Twitter, Facebook, or Skype. The online aspect allows researchers to tap into a large club where they can hear from and discuss with a wider peer group than may be possible locally. Previous work has indicated that journal clubs not only help researchers connect but also foster good research practices (Lizarondo etal. 2010).

One such club, Diversity Journal Club, is a multifaceted group, existing on a WordPress blog, and on Twitter, with their own hashtag #DiversityJC. The purpose of the group is to discuss diversity in STEM and academia, taking on a traditional “journal club” format, where they invite participants to discuss a particular article or blog post relating to diversity every month. Online forums such as journal clubs can be easy to join for short periods and get insight into literature that may be relevant to a researcher’s field.

#### Academic Blogs

Blogs fulfil several roles owing to the sheer diversity of blogs produced (Bukvova etal. 2010). As a free resource available to researchers, they can be a vital source of information and tips about research methods or even provide insight into research life (Reeve 2014). Although not as integrated as Facebook or Twitter, the comment sections on blog posts can also be a focus of interesting discussion and a place researchers can go to seek further advice and support. Blogs are also easy to set up through some of the platforms mentioned earlier, and many researchers may find it helpful to start their own as a way of reaching out for support. Examples of best practice in research blogs include *Bug Squad* (run by Garvey 2017, an entomologist from UC Davis) and *Don’t Forget About The Roundabouts* (run by Professor Leather 2017, an applied entomologist from Harper Adams University). There are a large number of blogs online, some more active than others, and finding a blog that gives a reader the information they are looking for can be difficult. There are some blog communities which bring together a number of blogs into one place such as *Occam*’*s Typewriter* and *Scientific American Blogs*.

### Advice

There are places on social media to specifically ask for advice from the community targeted in a similar way to research groups above. Typically, these are often areas within the more discussion-style social media and are places where research can post questions or share issues in their laboratory with other researchers. As these communities are based on the researchers within them, they can change frequently. The communities and tags listed below are a selection of the most consistently active groups.

#### Subreddits

As previously mentioned, there are subreddits within Reddit, which are communities aimed at providing a research or discussion area to post questions, queries, or rants on any given subject. One example is /r/AskAcademics, which is specifically geared toward academia. Another, /r/LabRats, has a broader remit for anyone working in research. Both have a strong community of active users from a wide range of backgrounds, who are always willing to share stories or advice. Researchers use subreddits to ask questions on topics spanning simple experiment advice to more serious allegations of misconduct. The anonymous nature of Reddit encourages open discussion both from the researchers asking questions and those replying. Users are not required to interact, so some may only add their voice to an existing issue, often providing broad discussion on a given topic. There are also some subject-specific subreddits such as/r/Entomology which, though typically consist of small communities, cater to questions and support within a specific field.

#### #PhDChat and #ECRChat

These are hashtags that are used on Facebook, Tumblr, and Twitter to connect early career researchers discussing research life. #PhDChat is much more commonly used and almost entirely comprised of other PhD students talking about their life and research during their PhD. #ECRChat has a similar aim, but is targeted at early career researchers (ECR), which typically consists of people in their first full time research position either as a postgraduate or postdoc. As communities based on hashtags, there is no “membership” as such, as people use it on an ad hoc basis. This results in a wide range of topics being discussed, and replies to specific questions can vary in quality and quantity depending on which researchers are online at the time. Despite this, the size of the community means that there is always ongoing discussion and frequently secondary hashtags for specific topics. There are also many subject-specific hashtags such as #entomology or #arachnology, which are often good places to look for advice on a niche topic.

#### #ICanHazPDF

#ICanHazPDF is a good example of a network specifically targeting a certain problem. In this case, the problem is the availability and access to research papers and literature. Many researchers, particularly those in industry, have limited access to research materials, and #ICanHazPDF is a community of researchers which actively seek out and share PDFs of these materials. On both Twitter and Facebook, posting a paper title or link followed by #ICanHazPDF will illicit replies from other academics with access to that paper, including either a unpaywalled link or a request for an e-mail address to send it to. Although this obviously has implications for copyright with journals, this is still an example of a thriving community of researchers helping each other out. In 2013, there were 10,000 tweets tagged with #ICanHazPDF (Liu 2013).

### Building Networks

In addition to the support and general advice groups, there are services specifically aimed not at resolving specific issues but instead promoting professional networking and engagement between researchers. Increased networking and engagement is particularly valuable to researchers looking to expand their contacts beyond those available through immediate peers. Social media also often provides a route to networking with researchers in other fields which users may not otherwise connect with.

#### RoCur

Rotational curation (RoCur) social media accounts are accounts that have a nominal subject which they discuss (such as biology or astrophysics), but are run by a rotating host. One of the largest examples is @RealScientists on Twitter, which has a different scientist running the account every week. Originally, RoCur was unique to Twitter but other platforms, such as Instagram, now also have rotational curation accounts. From a reader perspective, these offer insight into a wide range of other research activities and allow readers the opportunity to ask curators about their research. As a networking tool, though, RoCur is more effective when taking part as a curator. Being a curator allows researchers to show off their work to a wide audience, gaining immediate feedback and support. It also helps highlight them as a researcher and encourages people that may not have found them to follow and engage after their week on the account has finished. Some of the largest active research-orientated RoCur accounts are @RealScientists, @BioTweeps, @WeHumanities, @AstroTweeps, @IamSciComm, and @IAmSciArt.

#### Groups

As mentioned in section “Discussion Platforms,” many platforms offer the ability to form or join groups. These groups range from community-driven to professionally run and have a wide range of focuses. LinkedIn typically has groups aimed, and expanding users professionally network with researchers who can be of assistance. There are also networks aimed at connecting researchers with jobs. Facebook has a wider range of groups from local meetups (discussed in more detail below) to equipment sharing. Finding groups can be difficult because as they are easy to create, there are many to search through. Good groups to look for are ones specifically linked to a field and look for groups with the highest memberships, such as *TheEntomologyGroup* on Facebook or *Insects and Entomology* on LinkedIn. Some groups are also designed as “news feeds” of interesting research information and others encourage active engagement.

#### Meetups

Physical face-to-face meetups are the most active of all of the methods of improving researcher-isolation. Being face-to-face rather than online, they are not obviously linked to social media; however, we felt they should be included, as many researcher meetups are borne out of social media campaigns and discussions. With respect to research-isolation, meetups are particularly important because many of these social media-prompted meetups were created in a direct response to researcher isolation. On Twitter, these events are called “Tweetups” and can range in size from countrywide events to small local meetups of a couple of researchers. Many geographical regions have hashtags helping to connect people to the event (e.g., #CamSciTweepup) or specific groups researchers can join to get notified when there is an event in the local area (e.g., #UKSciMeetups). Typically these are community-driven and hosted on an ad hoc basis. There are groups dedicated to running regular meetups for scientists and actively encouraging them to come meet other researchers, as well as others working in science-related fields (e.g., London SciComm Socials). It is also becoming more common to run social media meetups as part of conferences, for example, an entomology “tweetup” at the International Congress of Entomology 2016 (Entomology Today 2016).

## Recommendations

As discussed in the introduction, this summary of social media networks is designed to give readers an insight into the plethora of networks and platforms available. Based on the discussion set out in this paper, we have two recommendations.

First, for any reader looking for social networking opportunities to combat isolation, our strongest recommendation is to try any of the mentioned networks. From our experience, every researcher has different needs and expectation of the support available, and none of the networks and communities discussed is a one-stop solution for everyone. We hope that the discussion and summary has provided enough information to choose a network that best suits the reader. For researchers who do not feel that the above choice of networks fulfils their needs, many of the projects and groups discussed were founded by researchers who also felt that they wanted a different kind of support, and so reached out to other researchers and formed the network they needed. Social media makes starting a network as easy as coining a new hashtag on one of the existing platforms. Guides such as *Ten Steps for Setting Up an Online Journal Club* (Chan etal. 2015) can provide a starting point for any researchers interested in starting their own network.

Second is a recommendation for academics who specialize in studying communities and mental health. The summary provided in this paper is based on all the available information that the authors could find through asking academics, social media experts, and journal searches. In the opinion of the authors, there is currently a worryingly small number of high quality peer-reviewed studies in the area of research isolation. It would be of great value to the research community to better understand the effect of these social media networks and their role in mental health and work satisfaction. There are signs that this need greater research is already being recognized with the launch of journals, such as Research for All, which focuses on the importance of public engagement in research, and we hope that in future more literature will be available to researchers wishing to make the best decisions about combating research-isolation.

Isolation is a difficult issue for many researchers around the world. Here, we have set out the roles that social media can play in providing support networks to those affected. There are a wide range of platforms and networks available for researchers, and we hope that by highlighting them in this paper, we have demonstrated the potential of social media in preventing and tackling isolation in academia.
